# Analysis of the association between CD40 and CD40 ligand polymorphisms and systemic sclerosis

**DOI:** 10.1186/ar3890

**Published:** 2012-06-25

**Authors:** María Teruel, Carmen P Simeon, Jasper Broen, Madelon C Vonk, Patricia Carreira, Maria Teresa Camps, Rosa García-Portales, Esmeralda Delgado-Frías, Maria Gallego, Gerard Espinosa, the Spanish Scleroderma Group, Lorenzo Beretta, Paolo Airó, Claudio Lunardi, Gabriela Riemekasten, Torsten Witte, Thomas Krieg, Alexander Kreuter, Jörg HW Distler, Nicolas Hunzelmann, Bobby P Koeleman, Alexandre E Voskuyl, Annemie J Schuerwegh, Miguel Ángel González-Gay, Timothy RDJ Radstake, Javier Martin

**Affiliations:** 1 Instituto de Parasitología y Biomedicina López-Neyra, IPBLN-CSIC, Avda. del Conocimiento s/n. 18010, Granada, SpainArmilla (Granada), Spain; 2 Department of Internal Medicine, Hospital Valle de Hebron, Passeig de la Vall d'Hebron 119 08035 Barcelona, Spain; 3 Department of Rheumatology, Radboud University Nijmegen Medical Centre, Comeniuslaan 4 6525 HP Nijmegen, The Netherlands; 4 Department of Rheumatology, Hospital 12 de Octubre, Avda. de Córdoba s/n 28041, Madrid, Spain; 5 Department of Internal Medicine, Hospital Carlos Haya, Avda Carlos Haya s/n 29010 Málaga, Spain; 6 Department of Rheumatology, Hospital Virgen de la Victoria, Campus de Teatinos s/n 29010 Málaga, Spain; 7 Department of Rheumatology, Hospital Universitario de Canarias, Ctra. Cuesta-Taco, s/n 38320, La Cuesta, San Cristóbal de La Laguna, Tenerife, Canarias, Spain; 8 Department of Internal Medicine, Hospital Central de Asturias, Celestino Villamil, s/n 33006 Oviedo, Spain; 9 Department of Autoimmune Diseases, Hospital Clinic, Carrer de Villarroel, 170 08036 Barcelona, Spain; 10 See Acknowledgements; 11 Referral Center for Systemic Autoimmune Diseases, Fondazione IRCCS Ca' Granda Ospedale Maggiore Policlinico Via Francesco Sforza, 35 20122 and University of Milan, Via Festa del Perdono, 7 20122, Milan, Italy; 12 Rheumatology Unit and Chair, Spedali Civili, Università degli Studi, Piazzale Spedali Civili, 1 25123 Brescia, Italy; 13 Department of Medicine, Policlinico GB Rossi, Università degli studi di Verona, Via dell'Artigliere, 8 37129 Verona, Italy; 14 Department of Rheumatology and Clinical Immunology, Charité University Hospital and German Rheumatism Research Centre, a Leibniz Institute, Charitéplatz 1, 10117 Berlin, Germany; 15 Clinic for Immunology and Rheumatology Medical School, Carl-Neuberg-Str, 1 30625, Hannover, Germany; 16 Department of Dermatology, University of Cologne, Kerpener Str 62, 50924 Köln, Germany; 17 Department of Dermatology, Allergology, and Venereology, Ruhr University of Bochum, Stiepeler Straße 129 44801 Bochum, Germany; 18 Department of Internal Medicine 3, Institute for Clinical Immunology, University of Erlangen-Nuremberg, Schillerstraße 1 91054, Erlangen, Germany; 19 Section Complex Genetics, Department of Medical Genetics, University Medical Center Utrecht, Universiteitsweg Stratenum 3508 AB, Utrecht, The Netherlands; 20 Department of Rheumatology, VU University Medical Center, De Boelelaan 1117 1081 HZ Amsterdam, The Netherlands; 21 Department of Rheumatology, Leiden University Medical Center, Albinusdreef 2 2300 RC, Leiden, The Netherlands; 22 Department of Rheumatology, Hospital Universitario Marques de Valdecilla, IFIMAV, Avda. Valdecilla, 25, 39008 Santander, Spain; 23 Department of Rheumatology and Clinical Immunology, University Utrecht Medical Center, Universiteitsweg 100 Stratenum 3508 AB, Utrecht, The Netherlands

## Abstract

**Introduction:**

The aim of the present study was to investigate the possible role of *CD40 *and *CD40 ligand (CD40LG) *genes in the susceptibility and phenotype expression of systemic sclerosis (SSc).

**Methods:**

In total, 2,670 SSc patients and 3,245 healthy individuals from four European populations (Spain, Germany, The Netherlands, and Italy) were included in the study. Five single-nucleotide polymorphisms (SNPs) of *CD40 *(rs1883832, rs4810485, rs1535045) and *CD40LG *(rs3092952, rs3092920) were genotyped by using a predesigned TaqMan allele-discrimination assay technology. Meta-analysis was assessed to determine whether an association exists between the genetic variants and SSc or its main clinical subtypes.

**Results:**

No evidence of association between *CD40 *and *CD40LG *genes variants and susceptibility to SSc was observed. Similarly, no significant statistical differences were observed when SSc patients were stratified by the clinical subtypes, the serologic features, and pulmonary fibrosis.

**Conclusions:**

Our results do not suggest an important role of *CD40 *and *CD40LG *gene polymorphisms in the susceptibility to or clinical expression of SSc.

## Introduction

Systemic sclerosis (SSc) is an autoimmune disease of the connective tissue characterized by excessive fibrosis of the dermis and vascular damage. It also affects internal organs, such as the lung, gastrointestinal, and vascular systems [1]. SSc is a complex polygenic disease in which environmental and genetic factors are involved in the susceptibility to this disease. Candidate gene and genome-wide association studies (GWASs) performed in SSc have identified new loci implicated in the susceptibility to SSc [2]. Nevertheless, the complete genetic components of SSc remain unknown.

CD40 is a member of the tumor necrosis factor receptor superfamily (TNFR), and it is expressed on the surface of several immune and nonhematopoietic cells, such as B cells, macrophages, dendritic cells, fibroblasts, and endothelial cells in certain pathogenic conditions [3]. Its ligand, CD40LG (CD154), is expressed mainly on the surface of CD4^+^ T cells. CD40-CD40LG interactions are necessary for the activation of both humoral and cellular immune responses [3]. The CD40-CD40LG pathway has been suggested to play an important role in the pathogenesis of autoimmune diseases [4]. An increase of soluble CD40LG (sCD40LG) has been observed in many autoimmune diseases, such as systemic lupus erythematosus (SLE) [5], rheumatoid arthritis (RA) [6], and Graves disease (GD) [7]. Interestingly, patients with SSc and limited cutaneous disease have higher levels of sCD40LG in the plasma than those of the diffuse cutaneous disease [8]. Similarly, high levels of CD40 protein were observed in both the plasma [9] and the cell surface of skin fibroblasts [10] from SSc patients. In addition, previous studies reported the association of *CD40 *polymorphisms with susceptibility to a number of autoimmune diseases, such as GD [11], multiple sclerosis [12], RA [13,14], Crohn disease [15], and with visual ischemic manifestations in individuals with biopsy-proven giant cell arteritis [16]. Nevertheless, in the case of SLE, contradictory data exist [17-19]. In addition, mutations of *CD40LG *were observed in patients with the hyper-immunoglobulin M (IgM) syndrome [20], and genetic variations located at the 3UTR of the *CD40LG *gene were associated with two autoimmune diseases, SLE [21] and RA [22].

Taking into account these considerations, we aimed to investigate the potential association of *CD40 *and *CD40LG *genes polymorphism with SSc.

## Materials and methods

### Patients

In total, 2,670 SSc patients and 3,245 healthy individuals from four European populations were included in this study (Spain: cases, 1,103; controls, 1,610; Germany: cases, 554; controls, 437; The Netherlands: cases, 380; controls, 489; Italy: cases, 633; controls, 709). All the patients fulfilled the 1980 American College of Rheumatology (ACR) classification criteria for SSc [23]. Limited cutaneous disease (lcSSc) was defined as definite skin thickening confined to the distal extremities, whereas cases of diffuse cutaneous disease (dcSSc) required also the involvement of skin proximal to the knees and elbow [24,25]. Measurement of main SSc-specific autoantibodies, anti-centromere antibodies (ACAs), and anti-topoisomerase I antibodies (ATAs), was performed by using standard methods. Pulmonary fibrosis data were investigated by using a computed tomography scan. The main features of all populations included in this study were previously reported [26-28].

Patients and controls were included in the study after written informed consent, according to the declaration of Helsinki. The study was approved by local ethical committees from all the participating centers.

### SNPs selection and genotyping

DNA from patients and controls was obtained by using standard methods. *CD40 *is located on chromosome 20q13.12. Three single-nucleotide polymorphisms (SNPs) of *CD40 *associated with other autoimmune diseases were selected. rs1883832 had been associated with GD [11]; whereas rs4810485, in linkage disequilibrium with rs1883832 (*r^2 ^*= 0.95), has been identified as a new risk factor for RA [13]. Furthermore, rs1535045 has been associated with subclinical atherosclerosis in diabetes families [29]. *CD40LG *is found on chromosome Xq26.3. Two genetic variants located in 5 UTR (rs3092952) and 3 UTR (rs3092920) of *CD40LG *(*r^2 ^*= 0.38) were selected. These SNPs are located in different haplotype blocks of *CD40LG *[17]. The variant rs3092920 is located near the 3 UTR microsatellite, which was previously associated with RA and SLE [21,22]; whereas rs3092952 is a functional variant related to the levels of sCD40LG in plasma [30].

All SNPs were genotyped in the same center by using a TaqMan SNP genotyping assay in a 7900HT Real-Time polymerase chain reaction (PCR) system, by following the conditions recommended by the manufacturer (Applied Biosystems, Foster City, CA, USA). About 10% of the patients were genotyped twice to verify the genotyping consistency, showing 99% identical genotypes. The genotyping call-rate success was >95% for both cases and controls in all populations.

### Statistical analysis

The Hardy-Weinberg equilibrium (HWE) was tested by means of the Fisher Exact test or χ^2^ when necessary. The case-control association study was performed by using a 2 × 2 contingency table with χ^2^ to obtain *P *values, odds ratios (ORs), and 95% confidence intervals (CIs). Combined OR was calculated according to a fixed-effects model (Mantel-Haenszel meta-analysis), and the heterogeneity of OR among all populations was calculated by using the Breslow-Day test. *P *values <0.05 were considered statistically significant. Statistical analyses were carried out with PLINK [31].

The estimation of the power of the study to detect an effect of a polymorphism in disease susceptibility was performed by using The CaTS Power Calculator software [32].

## Results

### *CD40 *gene variants in SSc

Three *CD40 *polymorphisms were genotyped in four European populations. First, we analyzed the cohorts individually and then combined the samples in a pooled analysis. Table 1 describes allelic distribution of the three SNPs in the pooled analysis, and Additional file 1, Table S1-3 contains detailed data for each population. Both cases and controls were confirmed to be in HWE in all populations. No evidence of association between *CD40 *polymorphisms and susceptibility to SSc was observed in the pooled analysis (allelic *P *value rs1883832: *P *= 0.61; OR, 1.02; 95% CI, 0.94 to 1.11; rs4810485: *P *= 0.42; OR, 1.04; 95% CI, 0.95 to 1.13; rs1535045: *P *= 0.275; OR, 0.95; 95% CI, 0.88 to 1.04).

**Table 1:** Pooled analysis of *CD40 *polymorphisms

SNP	Change	Samples set	*N*	Minor allele, no. (frequency)	*P *value	OR [95% CI]	*P-BD*
rs1883832	T/C	Controls	3,138	1,427 (0.277)			
		SSc	2,605	1,424 (0.274)	0.610	1.02 [0.94-1.11]	0.904
		lcSSc	1,758	987 (0.276)	0.246	1.06 [0.96-1.16]	0.496
		dcSSc	860	451 (0.271)	0.683	0.97 [0.86-1.10]	0.446
		ACA +	1,077	592 (0.261)	0.879	1.01 [0.90-1.13]	0.355
		ATA +	716	416 (0.293)	0.108	1.11 [0.98-1.26]	0.946
		Pulmonary fibrosis	702	394 (0.278)	0.433	1.05 [0.92-1.20]	0.802

rs4810485	G/T	Controls	3,122	1,652 (0.272)			
		SSc	2,560	1,388 (0.274)	0.420	1.04 [0.95-1.13]	0.935
		lcSSc	1,736	967 (0.277)	0.155	1.07 [0.97-1.18]	0.481
		dcSSc	839	436 (0.269)	0.817	0.99 [0.87-1.12]	0.600
		ACA +	1,071	580 (0.257)	0.844	1.01 [0.90-1.13]	0.399
		ATA +	699	402 (0.292)	0.080	1.12 [0.99-1.28]	0.890
		Pulmonary fibrosis	682	375 (0.272)	0.483	1.05 [0.92-1.20]	0.605

rs1535045	T/C	Controls	3,147	1,595 (0.247)			
		SSc	2,585	1,272 (0.243)	0.275	0.95 [0.88-1.04]	0.560
		lcSSc	1,749	860 (0.251)	0.375	0.96 [0.87-1.05]	0.334
		dcSSc	850	411 (0.228)	0.275	0.93 [0.82-1.06]	0.746
		ACA +	1,075	525 (0.241)	0.348	0.95 [0.84-1.06]	0.844
		ATA +	707	369 (0.264)	0.669	1.03 [0.90-1.18]	0.640
		Pulmonary fibrosis	696	340 (0.250)	0.396	0.94 [0.82-1.08]	0.028

Controls are used as reference for all comparisons. All *P *values have been calculated for the allelic model by using the Mantel-Haenszel test under fixed effect. P_BD, *P *value by the Breslow-Day method.

To investigate the possible influence of the *CD40 *polymorphisms with clinical features, we stratified the patients according to the main SSc manifestations. However, we did not observe evidence of association after comparing lcSSc and dcSSc with healthy subjects (see Table 1). Additionally, we compared the presence of SSc-specific autoantibodies in the patients with the healthy individuals, but no significant differences were observed (Table 1). Likewise, no significant association was observed when patients with pulmonary fibrosis were compared with healthy controls (Table 1).

### *CD40LG *gene variants in SSc

Because *CD40LG *is located on the X-chromosome and shows the sexual bias of this disease, we performed the analysis separately for each gender. Table 2 shows the allelic frequencies in SSc females of the two *CD40LG *polymorphisms in the pooled analysis, and Additional file 1, Table S4-5 shows the frequencies for each population. Deviations from HWE were not observed. With the Mantel-Haenzel test, the genotype and allele frequencies were similar between SSc patients and healthy individuals (allelic *P *value rs3092952: *P *= 0.44; OR, 0.96; 95% CI, 0.85 to 1.07; rs3092920: *P *= 0.565; OR, 0.96; 95% CI, 0.83 to 1.11). No statistical differences were observed when SSc patients were stratified by common subtype of the disease, the presence of SSc-specific autoantibodies, and pulmonary fibrosis (Table 2).

**Table 2:** Pooled analysis of *CD40LG *polymorphisms in female SSc patients and controls

SNP	Change	Samples set	*N*	Minor allele, no. (frequency)	*P *value	OR [95% CI]	*P*_BD
rs3092952	G/A	Controls	1,795	655 (0.176)			
		SSc	2,082	735 (0.186)	0.440	0.96 [0.85-1.07]	0.18
		lcSSc	1,446	515 (0.188)	0.592	0.97 [0.85-1.10]	0.12
		dcSSc	635	219 (0.179)	0.307	0.92 [0.77-1.09]	0.73
		ACA +	924	338 (0.195)	0.978	1.00 [0.87-1.16]	0.06
		ATA +	533	184 (0.188)	0.375	0.92 [0.77-1.11]	0.50
		Pulmonary fibrosis	542	198 (0.173)	0.735	0.97 [0.81-1.16]	0.41

rs3092920	T/G	Controls	1,789	376 (0.107)			
		SSc	2,104	426 (0.105)	0.565	0.96 [0.83-1.11]	0.89
		lcSSc	1,456	310 (0.112)	0.929	1.01 [0.86-1.18]	0.97
		dcSSc	647	116 (0.091)	0.120	0.84 [0.67-1.05]	0.36
		ACA +	939	201 (0.111)	0.924	1.01 [0.84-1.21]	0.48
		ATA +	541	106 (0.103)	0.434	0.91 [0.73-1.15]	0.86
		Pulmonary fibrosis	549	124 (0.106)	0.657	1.05 [0.84-1.31]	0.55

Controls are used as reference for all comparisons. All *P *values have been calculated for the allelic model by using the Mantel-Haenszel test under fixed effect. *P*_BD, *P *value by the Breslow-Day method.

In addition, we analyzed these two polymorphisms in SSc male patients, but no evidence of association was found in the combined analyses (see Additional file 1, Table S6-7). However, these results should be interpreted with caution because the statistical power is insufficient to detect association because of low sample size.

## Discussion

The important role of the CD40-CD40LG pathway in autoimmunity [4], together with the association of the *CD40 *gene with a number of autoimmune diseases, prompted us to investigate for the first time the contribution of *CD40 *and *CD40LG *genes in SSc.

Despite the previous findings [8-10], we observed no evidence of association of the *CD40 *or *CD40LG *gene variants analyzed with SSc. It was also the case when SSc patients were stratified by the SSc clinical subtypes, specific autoantibodies, or pulmonary fibrosis. We analyzed a large European population from four different countries to increase the robustness of the study. In this regard, our combined study had an estimated power of 92% to detect the relative risk, with OR of 1.15 obtained for RA susceptibility [13,14], at the 5% significance level. Therefore, it seems unlikely that the absence of association found in our study would be due to Type II error.

In the present study, we analyzed the functional *CD40*-1C/T polymorphism (rs1883832). This genetic variant is located at -1 from the ATG, within a Kozak sequence, a stretch of nucleotides essential for translation that flanks the start codon in vertebrate genes [33]. The presence of a major allele (C) in this SNP is associated with the increase of the efficiency of CD40 translations [11]. Although quantitative differences between CD40 mRNA and proteins have been observed in SSc skin fibroblasts [10], the absence of association found for rs1883832 suggests that this variant might not affect the translation of *CD40 *mRNA. This process may be upregulated in these abnormal skin fibroblasts, or other genes of the *CD40 *signaling pathway may influence the *CD40 *expression. However, functional studies in this way should be constructed before excluding an association between this variant and the *CD40 *expression in SSc.

Although *CD40 *might be a common susceptibility locus for some autoimmune diseases [11-16], our results do not suggest an important role of *CD40 *in the susceptibility to SSc. Several genes have been recently disclosed to play a function in the susceptibility to autoimmune diseases, suggesting that these diseases share a genetic background [34]. However, these loci may not be universal genetic factors for autoimmune disorders; therefore, the autoimmunity might result from specific and multiple pleiotropic effects [19]. Also, other genes are unique for each disease, reflecting a specific etiology [19,34]. Additional studies are required for the identification of specific and shared genetic pathways that contribute to a better understanding of the pathogenesis of the autoimmune diseases.

The role of the *CD40LG *in the susceptibility to autoimmune diseases has not been investigated as broadly as that of *CD40*, mainly because this gene is located on the × chromosome. The different prevalences of these diseases in both genders can suggest that genes located on the × chromosome could be susceptibility factors in autoimmune diseases; however, few studies analyzed polymorphisms on this chromosome. Mutations on this gene are associated with X-linked hyper-IgM syndrome, a familial genetic disorder characterized by an increase of IgM level and a decrease of IgG and IgA [20], but in SLE, no evidence of association has been found [17]. Similarly, our results show that the *CD40LG *gene may not be SSc susceptibility loci.

## Conclusions

Our results do not suggest an important role of *CD40 *and *CD40LG *genes in the susceptibility to SSc. Additional studies are required to draw firm conclusions about the exact role of the *CD40 *and *CD40LG *genes in SSc susceptibility because other variants might be involved in SSc. Future studies involving other genes of the CD40-CD40LG pathway should be conducted to elucidate fully the contribution of this pathway in the pathogenesis of SSc.

## Abbreviations

ACA: anti-centromere antibody; ATA: anti-topoisomerase I antibody; CI: confidence interval; dcSSc: diffuse cutaneous subtype; GD: Graves disease; GWAS: genome-wide association study; HWE: Hardy-Weinberg equilibrium; lcSSc: limited cutaneous subtype; OR: odds ratio; *P-*BD: *P *value by Breslow-Day method; RA: rheumatoid arthritis; SLE: systemic lupus erythematosus; SNP: single-nucleotide polymorphism; SSc: systemic sclerosis.

## Competing interests

The authors declare that they have no competing interests.

## Authors' contributions

MT and JM made substantial contributions to conception, design of study, and interpretation of data. MT carried out genotyping, analysis of data, and drafted the manuscript. CPS, JB, MCV, PC, MTC, RGP, EDF, MG, GE, LB, PA, CL, GR, TW, TK, AK, JHWD, NH, BPK, AEV, AJ, AJS, MAGG, TRDJR, and SSG had been involved in the acquisition of clinical data of the patients included in this study as well as the interpretation of the data. JM has been involved in revising of the final manuscript. All authors gave final approval of the version to be published.

## Supplementary Material

Additional file 1 **Supplementary Tables 1 through 7**. Genotype and allele distribution of the *CD40 *and *CD40LG *polymorphisms for each population included in the current study. Click here for file
