# Supplementary material for: Analysis of the association between CD40 and CD40 ligand polymorphisms and systemic sclerosis
Source: Arthritis Res Ther. 2012 Jun 25;14(3):R154. doi: 10.1186/ar3890 (PMC3446540; doi:10.1186/ar3890)
Supplement: Additional file 1 — Supplementary Tables 1 through 7. Genotype and allele distribution of the CD40 and CD40LG polymorphisms for each population included in the current study. [file ar3890-S1.DOC]

**CD40-CD40LG in SSc.**

**Additional file 1:** Supplementary tables 1-7

*Table S1*. Genotype and allele frequencies of *CD40* rs1883832 (-1C/T) polymorphism in the all populations included in the study.

| **Population** | **Samples Set** | **N** | **Genotype, no. (frequency)** | | | **Minor allele,** | **Allele test** | |
| --- | --- | --- | --- | --- | --- | --- | --- | --- |
|  |  |  | **C/C** | **C/T** | **T/T** | **no. (frequency)** | **P-value** | **OR [95% CI]** |
| Spain | Controls | 1546 | 815 (0.527) | 607 (0.393) | 124 (0.080) | 594 (0.274) |  |  |
|  | SSc | 1083 | 579 (0.535) | 414 (0.382) | 90 (0.083) | 594 (0.274) | 0.855 | 0.99 [0.87-1.12] |
|  | lcSSc | 735 | 393 (0.535) | 278 (0.378) | 64 (0.087) | 406 (0.276) | 0.981 | 1.00 [0.87-1.15] |
|  | dcSSc | 345 | 184 (0.533) | 135 (0.391) | 26 (0.075) | 187 (0.271) | 0.770 | 0.97 [0.81-1.17] |
|  | ACA + | 498 | 276 (0.554) | 184 (0.369) | 38 (0.076) | 260 (0.261) | 0.340 | 0.92 [0.79-1.09] |
|  | ATA + | 237 | 117 (0.494) | 101 (0.426) | 19 (0.080) | 139 (0.293) | 0.449 | 1.09 [0.88-1.34] |
|  | P. fibrosis | 250 | 132 (0.528) | 97 (0.388) | 21 (0.084) | 139 (0.278) | 0.945 | 1.01 [0.82-1.24] |
| Germany | Controls | 419 | 224 (0.535) | 174 (0.415) | 21 (0.050) | 216 (0.258) |  |  |
|  | SSc | 527 | 289 (0.548) | 190 (0.361) | 48 (0.091) | 286 (0.271) | 0.506 | 1.07 [0.87-1.32] |
|  | lcSSc | 318 | 166 (0.522) | 118 (0.371) | 34 (0.107) | 186 (0.293) | 0.152 | 1.18 [0.94-1.49] |
|  | dcSSc | 229 | 129 (0.563) | 85 (0.371) | 15 (0.066) | 115 (0.251) | 0.748 | 0.96 [0.74-1.24] |
|  | ACA + | 210 | 108 (0.514) | 79 (0.376) | 23 (0.110) | 125 (0.298) | 0.146 | 1.21 [0.94-1.57] |
|  | ATA + | 163 | 85 (0.521) | 68 (0.417) | 10 (0.061) | 88 (0.270) | 0.706 | 1.06 [0.79-1.41] |
|  | P. fibrosis | 170 | 84 (0.494) | 75 (0.441) | 11 (0.065) | 97 (0.285) | 0.356 | 1.14 [0.86-1.51] |
| The Netherland | Controls | 484 | 297 (0.614) | 156 (0.322) | 31 (0.064) | 218 (0.225) |  |  |
|  | SSc | 374 | 216 (0.578) | 142 (0.380) | 16 (0.043) | 174 (0.233) | 0.717 | 1.04 [0.83-1.31] |
|  | lcSSc | 255 | 137 (0.537) | 106 (0.416) | 12 (0.047) | 130 (0.255) | 0.201 | 1.18 [0.92-1.51] |
|  | dcSSc | 119 | 79 (0.664) | 36 (0.303) | 4 (0.034) | 44 (0.185) | 0.176 | 0.78 [0.54-1.12] |
|  | ACA + | 91 | 54 (0.593) | 34 (0.374) | 3 (0.033) | 40 (0.220) | 0.872 | 0.97 [0.66-1.42] |
|  | ATA + | 101 | 54 (0.535) | 44 (0.436) | 3 (0.030) | 50 (0.248) | 0.492 | 1.13 [0.79-1.61] |
|  | P. fibrosis | 84 | 47 (0.560) | 31 (0.369) | 6 (0.071) | 43 (0.256) | 0.382 | 1.18 [0.81-1.73] |
| Italy | Controls | 689 | 350 (0.508) | 279 (0.405) | 60 (0.087) | 399 (0.290) |  |  |
|  | SSc | 621 | 309 (0.498) | 254 (0.409) | 58 (0.093) | 370 (0.298) | 0.639 | 1.04 [0.88-1.23] |
|  | lcSSc | 450 | 230 (0.511) | 175 (0.389) | 45 (0.100) | 265 (0.294) | 0.802 | 1.02 [0.85-1.23] |
|  | dcSSc | 167 | 75 (0.449) | 79 (0.473) | 13 (0.078) | 105 (0.314) | 0.372 | 1.13 [0.87-1.46] |
|  | ACA + | 278 | 142 (0.511) | 105 (0.378) | 31 (0.112) | 167 (0.300) | 0.636 | 1.05 [0.85-1.31] |
|  | ATA + | 215 | 96 (0.447) | 99 (0.460) | 20 (0.093) | 139 (0.323) | 0.182 | 1.17 [0.93-1.48] |
|  | P. fibrosis | 198 | 102 (0.515) | 77 (0.389) | 19 (0.096) | 115 (0.290) | 0.974 | 1.00 [0.79-1.29] |

*Table S2*. Genotype and allele frequencies of *CD40* rs4810485 (G/T) polymorphism in the all populations included in the study.

|  |  |  | **Genotype, no. (frequency)** | | | **Minor allele,** | **Allele test** | |
| --- | --- | --- | --- | --- | --- | --- | --- | --- |
| **Population** | **Samples Set** | **N** | **G/G** | **G/T** | **T/T** | **no. (frequency)** | **P-value** | **OR [95% CI]** |
| Spain | Controls | 1549 | 829 (0.535) | 597 (0.385) | 123 (0.079) | 843 (0.272) |  |  |
|  | SSc | 1057 | 566 (0.535) | 402 (0.380) | 89 (0.084) | 580 (0.274) | 0.858 | 1.01 [0.89-1.15] |
|  | lcSSc | 722 | 386 (0.535) | 272 (0.377) | 64 (0.089) | 400 (0.277) | 0.730 | 1.03 [0.89-1.18] |
|  | dcSSc | 334 | 179 (0.536) | 130 (0.389) | 25 (0.075) | 180 (0.270) | 0.889 | 0.99 [0.82-1.19] |
|  | ACA + | 498 | 280 (0.562) | 180 (0.361) | 38 (0.076) | 256 (0.257) | 0.350 | 0.93 [0.79-1.09] |
|  | ATA + | 226 | 112 (0.496) | 96 (0.425) | 18 (0.080) | 132 (0.292) | 0.375 | 1.10 [0.89-1.37] |
|  | P. fibrosis | 239 | 129 (0.540) | 90 (0.377) | 20 (0.084) | 130 (0.272) | 0.995 | 1.00 [0.80-1.24] |
| Germany | Controls | 411 | 223 (0.543) | 167 (0.406) | 21 (0.051) | 209 (0.254) |  |  |
|  | SSc | 516 | 289 (0.560) | 179 (0.347) | 48 (0.093) | 275 (0.267) | 0.552 | 1.07 [0.86-1.31] |
|  | lcSSc | 311 | 166 (0.534) | 111 (0.357) | 34 (0.109) | 179 (0.288) | 0.172 | 1.18 [0.93-1.48] |
|  | dcSSc | 225 | 129 (0.573) | 81 (0.360) | 15 (0.067) | 111 (0.247) | 0.717 | 0.95 [0.73-1.24] |
|  | ACA + | 204 | 108 (0.529) | 73 (0.358) | 23 (0.113) | 119 (0.292) | 0.179 | 1.20 [0.92-1.56] |
|  | ATA + | 161 | 86 (0.534) | 65 (0.404) | 10 (0.062) | 85 (0.264) | 0.776 | 1.04 [0.78-1.40] |
|  | P. fibrosis | 165 | 85 (0.515) | 69 (0.418) | 11 (0.067) | 91 (0.276) | 0.484 | 1.11 [0.83-1.47] |
| The Netherland | Controls | 480 | 296 (0.617) | 154 (0.321) | 30 (0.063) | 214 (0.223) |  |  |
|  | SSc | 371 | 210 (0.566) | 145 (0.391) | 16 (0.043) | 177 (0.239) | 0.447 | 1.09 [0.87-1.37] |
|  | lcSSc | 254 | 134 (0.528) | 108 (0.425) | 12 (0.047) | 132 (0.260) | 0.113 | 1.22 [0.95-1.57] |
|  | dcSSc | 117 | 76 (0.650) | 37 (0.316) | 4 (0.034) | 45 (0.192) | 0.308 | 0.83 [0.58-1.19] |
|  | ACA + | 90 | 53 (0.589) | 34 (0.378) | 3 (0.033) | 40 (0.222) | 0.984 | 1.00 [0.68-1.46] |
|  | ATA + | 101 | 52 (0.515) | 45 (0.446) | 4 (0.040) | 53 (0.262) | 0.226 | 1.24 [0.88-1.76] |
|  | P. fibrosis | 81 | 43 (0.531) | 32 (0.395) | 6 (0.074) | 44 (0.272) | 0.173 | 1.30 [0.89-1.90] |
| Italy | Controls | 682 | 352 (0.516) | 274 (0.402) | 56 (0.082) | 386 (0.283) |  |  |
|  | SSc | 616 | 316 (0.513) | 244 (0.396) | 56 (0.091) | 356 (0.289) | 0.737 | 1.03 [0.87-1.22] |
|  | lcSSc | 449 | 237 (0.528) | 168 (0.374) | 44 (0.098) | 256 (0.285) | 0.914 | 1.01 [0.84-1.22] |
|  | dcSSc | 163 | 75 (0.460) | 76 (0.466) | 12 (0.074) | 100 (0.307) | 0.395 | 1.12 [0.86-1.46] |
|  | ACA + | 279 | 144 (0.516) | 105 (0.376) | 30 (0.108) | 165 (0.296) | 0.576 | 1.06 [0.86-1.32] |
|  | ATA + | 211 | 99 (0.469) | 92 (0.436) | 20 (0.095) | 132 (0.313) | 0.238 | 1.15 [0.91-1.46] |
|  | P. fibrosis | 197 | 105 (0.533) | 74 (0.376) | 18 (0.091) | 110 (0.279) | 0.883 | 0.98 [0.76-1.26] |

*Table S3*. Genotype and allele frequencies of *CD40* rs1535045 (C/T) polymorphism in the all populations included in the study.

|  |  |  | **Genotype, no. (frequency)** | | | **Minor allele,** | **Allele test** | |
| --- | --- | --- | --- | --- | --- | --- | --- | --- |
| **Population** | **Samples Set** | **N** | **C/C** | **C/T** | **T/T** | **no. (frequency)** | **P-value** | **OR [95% CI]** |
| Spain | Controls | 1559 | 887 (0.569) | 573 (0.368) | 99 (0.064) | 771 (0.247) |  |  |
|  | SSc | 1060 | 613 (0.578) | 379 (0.358) | 68 (0.064) | 515 (0.243) | 0.720 | 0.98 [0.86-1.11] |
|  | lcSSc | 720 | 408 (0.567) | 263 (0.365) | 49 (0.068) | 361 (0.251) | 0.804 | 1.02 [0.88-1.18] |
|  | dcSSc | 338 | 203 (0.601) | 116 (0.343) | 19 (0.056) | 154 (0.228) | 0.285 | 0.90 [0.74-1.09] |
|  | ACA + | 492 | 278 (0.565) | 191 (0.388) | 23 (0.047) | 237 (0.241) | 0.683 | 0.97 [0.82-1.14] |
|  | ATA + | 229 | 123 (0.537) | 91 (0.397) | 15 (0.066) | 121 (0.264) | 0.435 | 1.09 [0.87-1.37] |
|  | P. fibrosis | 242 | 141 (0.583) | 81 (0.335) | 20 (0.083) | 121 (0.250) | 0.897 | 1.02 [0.81-1.27] |
| Germany | Controls | 417 | 222 (0.532) | 163 (0.391) | 32 (0.077) | 227 (0.272) |  |  |
|  | SSc | 526 | 289 (0.549) | 209 (0.397) | 28 (0.053) | 265 (0.252) | 0.319 | 0.90 [0.73-1.11] |
|  | lcSSc | 318 | 178 (0.560) | 122 (0.384) | 18 (0.057) | 158 (0.248) | 0.382 | 0.90 [0.71-1.14] |
|  | dcSSc | 228 | 128 (0.561) | 90 (0.395) | 10 (0.044) | 110 (0.241) | 0.283 | 0.87 [0.67-1.13] |
|  | ACA + | 210 | 118 (0.562) | 80 (0.381) | 12 (0.057) | 104 (0.248) | 0.426 | 0.90 [0.69-1.17] |
|  | ATA + | 161 | 88 (0.547) | 62 (0.385) | 11 (0.068) | 84 (0.261) | 0.793 | 0.96 [0.72-1.29] |
|  | P. fibrosis | 170 | 102 (0.600) | 62 (0.365) | 6 (0.035) | 74 (0.218) | 0.068 | 0.76 [0.56-1.02] |
| The Netherland | Controls | 483 | 274 (0.567) | 174 (0.360) | 35 (0.072) | 244 (0.253) |  |  |
|  | SSc | 375 | 229 (0.611) | 125 (0.333) | 21 (0.056) | 167 (0.223) | 0.150 | 0.85 [0.68-1.06] |
|  | lcSSc | 257 | 163 (0.634) | 80 (0.311) | 14 (0.054) | 108 (0.210) | 0.068 | 0.79 [0.61-1.02] |
|  | dcSSc | 118 | 66 (0.559) | 45 (0.381) | 7 (0.059) | 59 (0.250) | 0.935 | 0.99 [0.71-1.37] |
|  | ACA + | 91 | 58 (0.637) | 26 (0.286) | 7 (0.077) | 40 (0.220) | 0.347 | 0.83 [0.57-1.22] |
|  | ATA + | 102 | 59 (0.578) | 40 (0.392) | 3 (0.029) | 46 (0.226) | 0.415 | 0.86 [0.60-1.23] |
|  | P. fibrosis | 83 | 59 (0.711) | 19 (0.229) | 5 (0.060) | 29 (0.175) | 0.030 | 0.63 [0.41-0.96] |
| Italy | Controls | 688 | 372 (0.541) | 279 (0.406) | 37 (0.054) | 353 (0.257) |  |  |
|  | SSc | 624 | 333 (0.534) | 257 (0.412) | 34 (0.054) | 325 (0.260) | 0.821 | 1.02 [0.86-1.22] |
|  | lcSSc | 454 | 247 (0.544) | 181 (0.399) | 26 (0.057) | 233 (0.257) | 0.997 | 1.00 [0.83-1.21] |
|  | dcSSc | 166 | 86 (0.518) | 72 (0.434) | 8 (0.048) | 88 (0.265) | 0.750 | 1.05 [0.80-1.37] |
|  | ACA + | 282 | 152 (0.539) | 116 (0.411) | 14 (0.050) | 144 (0.255) | 0.955 | 0.99 [0.79-1.24] |
|  | ATA + | 215 | 110 (0.512) | 92 (0.428) | 13 (0.060) | 118 (0.274) | 0.461 | 1.10 [0.86-1.40] |
|  | P. fibrosis | 201 | 100 (0.498) | 86 (0.428) | 15 (0.075) | 116 (0.289) | 0.200 | 1.18 [0.92-1.51] |

*Table S4*. Genotype and allele distribution of the *CD40LG* rs3092952 (A/G) polymorphism in SSc females of the populations included in the study.

|  |  |  | **Genotype, no. (frequency) CASES** | | | **Minor allele,** | **Allele test** | |
| --- | --- | --- | --- | --- | --- | --- | --- | --- |
| **Populations** | **Samples Set** | **N** | **A/A** | **A/G** | **G/G** | **no. (frequency)** | **P-value** | **OR [95% CI]** |
| Spain | Controls | 890 | 610 (0.685) | 246 (0.276) | 34 (0.038) | 314 (0.176) |  |  |
|  | SSc | 910 | 613 (0.674) | 256 (0.281) | 41 (0.045) | 338 (0.186) | 0.468 | 1.07 [0.90-1.26] |
|  | lcSSc | 632 | 422 (0.668) | 182 (0.288) | 28 (0.044) | 238 (0.188) | 0.402 | 1.08 [0.90-1.31] |
|  | dcSSc | 277 | 191 (0.690) | 73 (0.264) | 13 (0.047) | 99 (0.179) | 0.902 | 1.02 [0.79-1.30] |
|  | ACA + | 429 | 281 (0.655) | 129 (0.301) | 19 (0.044) | 167 (0.195) | 0.256 | 1.13 [0.92-1.39] |
|  | ATA + | 194 | 128 (0.660) | 59 (0.304) | 7 (0.036) | 73 (0.188) | 0.584 | 1.08 [0.82-1.44] |
|  | P. Fibrosis + | 202 | 144 (0.713) | 46 (0.228) | 12 (0.059) | 70 (0.173) | 0.881 | 0.98 [0.74-1.30] |
| Germany | Controls | 221 | 142 (0.643) | 66 (0.299) | 13 (0.059) | 92 (0.208) |  |  |
|  | SSc | 420 | 294 (0.700) | 112 (0.267) | 14 (0.033) | 140 (0.167) | 0.067 | 0.76 [0.57-1.02] |
|  | lcSSc | 267 | 190 (0.712) | 70 (0.262) | 7 (0.026) | 84 (0.157) | 0.120* | 0.71 [0.51-0.98] |
|  | dcSSc | 153 | 104 (0.680) | 42 (0.275) | 7 (0.046) | 56 (0.183) | 0.396 | 0.85 [0.59-1.23] |
|  | ACA + | 181 | 133 (0.735) | 44 (0.243) | 4 (0.022) | 52 (0.144) | 0.027* | 0.64 [0.44-0.93] |
|  | ATA + | 109 | 76 (0.697) | 29 (0.266) | 4 (0.037) | 37 (0.170) | 0.242 | 0.78 [0.51-1.19] |
|  | P. Fibrosis | 123 | 78 (0.634) | 40 (0.325) | 5 (0.041) | 50 (0.203) | 0.879 | 0.97 [0.66-1.43] |
| The Netherland | Controls | 267 | 183 (0.685) | 74 (0.277) | 10 (0.037) | 94 (0.176) |  |  |
|  | *SSc | 228 | 170 (0.746) | 49 (0.215) | 9 (0.039) | 67 (0.147) | 0.216 | 0.81 [0.57-1.14] |
|  | lcSSc | 159 | 120 (0.755) | 31 (0.195) | 8 (0.050) | 47 (0.148) | 0.284 | 0.81 [0.55-1.19] |
|  | dcSSc | 69 | 50 (0.725) | 18 (0.261) | 1 (0.014) | 20 (0.145) | 0.386 | 0.79 [0.47-1.34] |
|  | ACA + | 58 | 38 (0.655) | 17 (0.293) | 3 (0.052) | 23 (0.198) | 0.572 | 1.16 [0.70-1.92] |
|  | ATA + | 57 | 42 (0.737) | 14 (0.246) | 1 (0.018) | 16 (0.140) | 0.357 | 0.76 [0.43-1.36] |
|  | P. Fibrosis | 53 | 42 (0.792) | 10 (0.189) | 1 (0.019) | 12 (0.113) | 0.112 | 0.60 [0.31-1.13] |
| Italy | Controls | 417 | 283 (0.679) | 113 (0.271) | 21 (0.050) | 155 (0.186) |  |  |
|  | SSc | 524 | 352 (0.672) | 154 (0.294) | 18 (0.034) | 190 (0.181) | 0.800 | 0.97 [0.77-1.23] |
|  | lcSSc | 388 | 258 (0.665) | 114 (0.294) | 16 (0.041) | 146 (0.188) | 0.906 | 1.02 [0.79-1.30] |
|  | dcSSc | 136 | 94 (0.691) | 40 (0.294) | 2 (0.015) | 44 (0.162) | 0.369 | 0.85 [0.59-1.22] |
|  | ACA + | 256 | 171 (0.668) | 74 (0.289) | 11 (0.043) | 96 (0.188) | 0.940 | 1.01 [0.76-1.34] |
|  | ATA + | 173 | 120 (0.694) | 48 (0.277) | 5 (0.029) | 58 (0.168) | 0.459 | 0.88 [0.63-1.23] |
|  | P. Fibrosis | 164 | 105 (0.640) | 52 (0.317) | 7 (0.043) | 66 (0.201) | 0.548 | 1.10 [0.80-1.52] |

* *P*-value corrected by FDR.

*Table S5*. Genotype and allele distribution of the *CD40LG* rs3092920 (G/T) polymorphism in SSc females of the populations included in the study.

|  |  |  | **Genotype, no. (frequency)** | | | **Minor allele,** | **Allele test** | |
| --- | --- | --- | --- | --- | --- | --- | --- | --- |
| **Populations** | **Samples Set** | **N** | **G/G** | **G/T** | **T/T** | **no. (frequency)** | **P-value** | **OR [95% CI]** |
| Spain | Controls | 879 | 707 (0.804) | 156 (0.177) | 16 (0.018) | 188 (0.107) |  |  |
|  | SSc | 907 | 730 (0.805) | 163 (0.180) | 14 (0.015) | 191 (0.105) | 0.873 | 0.98 [0.79-1.22] |
|  | lcSSc | 627 | 498 (0.794) | 118 (0.188) | 11 (0.018) | 140 (0.112) | 0.683 | 1.05 [0.83-1.32] |
|  | dcSSc | 279 | 231 (0.828) | 45 (0.161) | 3 (0.011) | 51 (0.091) | 0.293 | 0.84 [0.61-1.16] |
|  | ACA + | 431 | 343 (0.796) | 80 (0.186) | 8 (0.019) | 96 (0.111) | 0.732 | 1.05 [0.81-1.36] |
|  | ATA + | 195 | 156 (0.800) | 38 (0.195) | 1 (0.005) | 40 (0.103) | 0.800 | 0.95 [0.67-1.37] |
|  | P. Fibrosis | 203 | 166 (0.818) | 31 (0.153) | 6 (0.030) | 43 (0.106) | 0.952 | 0.99 [0.70-1.40] |
| Germany | Controls | 221 | 181 (0.819) | 36 (0.163) | 4 (0.018) | 44 (0.100) |  |  |
|  | SSc | 434 | 360 (0.829) | 68 (0.157) | 6 (0.014) | 80 (0.092) | 0.666 | 0.92 [0.62-1.35] |
|  | lcSSc | 272 | 224 (0.824) | 45 (0.165) | 3 (0.011) | 51 (0.094) | 0.759 | 0.94 [0.61-1.43] |
|  | dcSSc | 162 | 136 (0.840) | 23 (0.142) | 3 (0.019) | 29 (0.090) | 0.640 | 0.89 [0.54-1.46] |
|  | ACA + | 188 | 158 (0.840) | 28 (0.149) | 2 (0.011) | 32 (0.085) | 0.478 | 0.84 [0.52-1.36] |
|  | ATA + | 113 | 95 (0.841) | 16 (0.142) | 2 (0.018) | 20 (0.089) | 0.646 | 0.88 [0.50-1.53] |
|  | P. Fibrosis | 125 | 95 (0.760) | 27 (0.216) | 3 (0.024) | 33 (0.132) | 0.192 | 1.38 [0.85-2.23] |
| The Netherland | Controls | 263 | 225 (0.856) | 35 (0.133) | 3 (0.011) | 41 (0.078) |  |  |
|  | SSc | 232 | 197 (0.849) | 31 (0.134) | 4 (0.017) | 39 (0.084) | 0.725 | 1.09 [0.69-1.72] |
|  | lcSSc | 162 | 141 (0.870) | 17 (0.105) | 4 (0.025) | 25 (0.077) | 0.967 | 0.99 [0.59-1.66] |
|  | dcSSc | 70 | 56 (0.800) | 14 (0.200) | 0 (0.000) | 14 (0.100) | 0.400 | 1.31 [0.69-2.49] |
|  | ACA + | 61 | 49 (0.803) | 10 (0.164) | 2 (0.033) | 14 (0.115) | 0.189 | 1.53 [0.81-2.91] |
|  | ATA + | 56 | 46 (0.821) | 10 (0.179) | 0 (0.000) | 10 (0.089) | 0.688 | 1.16 [0.56-2.39] |
|  | P. Fibrosis | 53 | 48 (0.906) | 4 (0.075) | 1 (0.019) | 6 (0.057) | 0.445 | 0.71 [0.29-1.72] |
| Italy | Controls | 426 | 333 (0.782) | 83 (0.195) | 10 (0.023) | 103 (0.121) |  |  |
|  | SSc | 531 | 417 (0.785) | 112 (0.211) | 2 (0.004) | 116 (0.109) | 0.426 | 0.89 [0.67-1.18] |
|  | lcSSc | 395 | 303 (0.767) | 90 (0.228) | 2 (0.005) | 94 (0.119) | 0.906 | 0.98 [0.73-1.32] |
|  | dcSSc | 136 | 114 (0.838) | 22 (0.162) | 0 (0.000) | 22 (0.081) | 0.068 | 0.64 [0.40-1.04] |
|  | ACA + | 259 | 200 (0.772) | 59 (0.228) | 0 (0.000) | 59 (0.114) | 0.698 | 0.93 [0.67-1.31] |
|  | ATA + | 177 | 143 (0.808) | 32 (0.181) | 2 (0.011) | 36 (0.102) | 0.342 | 0.82 [0.55-1.23] |
|  | P. Fibrosis | 168 | 127 (0.756) | 40 (0.238) | 1 (0.006) | 42 (0.125) | 0.846 | 1.04 [0.71-1.52] |

*Table S6*. Genotype and allele frequencies of *CD40LG* rs3092952 (A/G) polymorphism in the SSc males of the populations included in the study and the pooled-analysis.

|  |  |  | **Minor allele,** | **Allele test** | |  |
| --- | --- | --- | --- | --- | --- | --- |
| **Populations** | **Samples Set** | **N** | **no. (frequency)** | **P-value** | **OR [95% CI]** | **PBD** |
| Spain | Controls | 677 | 108 (0.167) |  |  |  |
|  | SSc | 136 | 20 (0.165) | 0.954 | 0.98 [0.58-1.66] |  |
|  | lcSSc | 80 | 8 (0.111) | 0.218 | 0.62 [0.29-1.33] |  |
|  | dcSSc | 54 | 11 (0.229) | 0.274 | 1.48 [0.73-2.99] |  |
|  | ACA + | 55 | 7 (0.149) | 0.742 | 0.87 [0.38-1.99] |  |
|  | ATA + | 30 | 9 (0.333) | 0.052* | 2.49 [1.09-5.68] |  |
|  | Pulmonary fibrosis | 40 | 5 (0.152) | 0.811 | 0.89 [0.34-2.35] |  |
| Germany | Controls | 196 | 28 (0.151) |  |  |  |
|  | SSc | 84 | 14 (0.180) | 0.557 | 1.23 [0.61-2.50] |  |
|  | lcSSc | 32 | 4 (0.143) | 0.915 | 0.94 [0.30-2.92] |  |
|  | dcSSc | 52 | 10 (0.200) | 0.398 | 1.41 [0.63-3.14] |  |
|  | ACA + | 16 | 1 (0.083) | 0.523 | 0.51 [0.06-4.13] |  |
|  | ATA + | 37 | 8 (0.222) | 0.286 | 1.61 [0.67-3.90] |  |
|  | Pulmonary fibrosis | 39 | 7 (0.184) | 0.602 | 1.27 [0.51-3.18] |  |
| Holland | Controls | 171 | 26 (0.153) |  |  |  |
|  | SSc | 100 | 16 (0.163) | 0.823 | 1.08 [0.55-2.13] |  |
|  | lcSSc | 62 | 12 (0.197) | 0.429 | 1.36 [0.64-2.89] |  |
|  | dcSSc | 38 | 4 (0.108) | 0.483 | 0.67 [0.22-2.06] |  |
|  | ACA + | 12 | 2 (0.167) | 0.899 | 1.11 [0.23-5.35] |  |
|  | ATA + | 40 | 5 (0.125) | 0.654 | 0.79 [0.28-2.21] |  |
|  | Pulmonary fibrosis | 29 | 9 (0.321) | 0.046* | 2.62 [1.07-6.43] |  |
| Italy | Controls | 240 | 33 (0.148) |  |  |  |
|  | SSc | 50 | 4 (0.085) | 0.255 | 0.54 [0.18-1.59] |  |
|  | lcSSc | 30 | 4 (0.143) | 0.943 | 0.96 [0.31-2.95] |  |
|  | dcSSc | 20 | 0 (0.000) | 0.071 | - |  |
|  | ACA + | 6 | 1 (0.200) | 0.747 | 1.44 [0.16-13.28] |  |
|  | ATA + | 0 | 0 (0.000) | 0.095* | - |  |
|  | Pulmonary fibrosis | 0 | 0 (0.000) | 0.052 | - |  |
| Pooled | Controls | 1284 | 195 (0.159) |  |  |  |
|  | SSc | 370 | 54 (0.157) | 0.942 | 0.99 [0.71-1.38] | 0.637 |
|  | lcSSc | 204 | 28 (0.148) | 0.665 | 0.91 [0.58-1.41] | 0.560 |
|  | dcSSc | 164 | 25 (0.162) | 0.857 | 1.04 [0.66-1.66] | 0.139 |
|  | ACA + | 89 | 11 (0.145) | 0.708 | 0.88 [0.46-1.70] | 0.911 |
|  | ATA + | 107 | 22 (0.175) | 0.515 | 1.18 [0.72-1.93] | 0.030 |
|  | Fibrosis + | 108 | 21 (0.174) | 0.664 | 1.12 [0.68-1.86] | 0.044 |

* *P*-value corrected by FDR.

PBD: P-value by Breslow-Day method

*Table S7*. Genotype and allele distribution of the *CD40LG* rs3092920 (G/T) polymorphism in the SSc males of the populations included in the study and the pooled-analysis.

|  |  |  | **Minor allele,** | **Allele test** | |  |
| --- | --- | --- | --- | --- | --- | --- |
| **Populations** | **Samples Set** | **N** | **no. (frequency)** | **P-value** | **OR [95% CI]** | **PBD** |
| Spain | Controls | 677 | 82 (0.127) |  |  |  |
|  | SSc | 136 | 9 (0.072) | 0.080 | 0.53 [0.26-1.09] |  |
|  | lcSSc | 80 | 5 (0.068) | 0.145 | 0.50 [0.20-1.29] |  |
|  | dcSSc | 54 | 4 (0.078) | 0.307 | 0.58 [0.20-1.66] |  |
|  | ACA + | 55 | 5 (0.102) | 0.607 | 0.78 [0.30-2.02] |  |
|  | ATA + | 30 | 3 (0.103) | 0.705 | 0.79 [0.23-2.67] |  |
|  | Pulmonary fibrosis | 40 | 2 (0.056) | 0.203 | 0.40 [0.10-1.71] |  |
| Germany | Controls | 196 | 16 (0.084) |  |  |  |
|  | SSc | 84 | 8 (0.099) | 0.690 | 1.20 [0.49-2.92] |  |
|  | lcSSc | 32 | 2 (0.065) | 0.716 | 0.75 [0.16-3.45] |  |
|  | dcSSc | 52 | 6 (0.120) | 0.428 | 1.49 [0.55-4.03] |  |
|  | ACA + | 16 | 1 (0.067) | 0.817 | 0.78 [0.10-6.33] |  |
|  | ATA + | 37 | 5 (0.139) | 0.295 | 1.76 [0.60-5.17] |  |
|  | Pulmonary fibrosis | 39 | 5 (0.135) | 0.323 | 1.71 [0.58-5.00] |  |
| Holland | Controls | 171 | 10 (0.059) |  |  |  |
|  | SSc | 100 | 9 (0.095) | 0.277 | 1.67 [0.66-4.28] |  |
|  | lcSSc | 62 | 6 (0.100) | 0.281 | 1.78 [0.62-5.12] |  |
|  | dcSSc | 38 | 3 (0.086) | 0.552 | 1.50 [0.39-5.76] |  |
|  | ACA + | 12 | 1 (0.083) | 0.731 | 1.46 [0.17-12.42] |  |
|  | ATA + | 40 | 3 (0.079) | 0.643 | 1.37 [0.36-5.24] |  |
|  | Pulmonary fibrosis | 29 | 6 (0.240) | 0.004* | 5.05 [1.65-15.46] |  |
| Italy | Controls | 240 | 22 (0.096) |  |  |  |
|  | SSc | 50 | 3 (0.063) | 0.461 | 0.63 [0.18-2.19] |  |
|  | lcSSc | 30 | 3 (0.103) | 0.899 | 1.09 [0.30-3.88] |  |
|  | dcSSc | 20 | 0 (0.000) | 0.157 | - |  |
|  | ACA + | 6 | 1 (0.200) | 0.440 | 2.35 [0.25-21.98] |  |
|  | ATA + | 0 | 0 (0.000) | 0.112 | - |  |
|  | Pulmonary fibrosis | 0 | 0 (0.000) | 0.128 | - |  |
| Pooled | Controls | 1284 | 130 (0.105) |  |  |  |
|  | SSc | 370 | 29 (0.831) | 0.393 | 0.83 [0.54-1.27] | 0.204 |
|  | lcSSc | 204 | 16 (0.083) | 0.531 | 0.84 [0.48-1.46] | 0.340 |
|  | dcSSc | 164 | 13 (0.084) | 0.601 | 0.85 [0.46-1.56] | 0.244 |
|  | ACA + | 89 | 8 (0.099) | 0.821 | 0.92 [0.43-1.95] | 0.789 |
|  | ATA + | 107 | 11 (0.087) | 0.881 | 0.95 [0.49-1.84] | 0.252 |
|  | Fibrosis + | 108 | 13 (0.108) | 0.706 | 1.13 [0.61-2.08] | 0.004 |

* *P*-value corrected by FDR.

PBD: P-value by Breslow-Day method
